# Butyrate inhibits pro-proliferative miR-92a by diminishing c-Myc-induced miR-17-92a cluster transcription in human colon cancer cells

**DOI:** 10.1186/s12943-015-0450-x

**Published:** 2015-10-13

**Authors:** Shien Hu, Lan Liu, Eugene B. Chang, Jian-Ying Wang, Jean-Pierre Raufman

**Affiliations:** VA Maryland Healthcare System, Department of Medicine, Division of Gastroenterology & Hepatology, and the Marlene and Stewart Greenebaum Cancer Center, University of Maryland School of Medicine, 22 South Greene Street, N3W62, Baltimore, MD 21201 USA; VA Maryland Healthcare System, Department of Surgery, University of Maryland School of Medicine, Baltimore, MD USA; The Martin Boyer Laboratories, Department of Medicine, University of Chicago School of Medicine, Chicago, IL USA

**Keywords:** Butyrate, c-Myc, miR-92a, HDAC inhibitor, miRNA, p57, Colon cancer

## Abstract

**Background:**

Compromised colonic butyrate production resulting from low dietary fiber or altered gut microbiota may promote colon neoplasia. Previous reports indicate these actions are mediated in part by altered levels of miRNAs, including suppressed expression of the oncogenic miR-17-92a cluster. Here, we sought to identify the mechanisms underlying these effects of butyrate in colon cancer.

**Methods:**

miR-92a levels were measured in archived human colon cancer and adjacent normal colon specimens by microarray and quantitative RT-PCR (qPCR). The effects of butyrate and other histone deacetylase inhibitors (suberoylanilide hydroxamic acid (SAHA) and valproic acid) on primary (pri-miR17-92a), precursor and mature miR-92a were analyzed in HCT-116 and HT-29 human colon cancer cells using qPCR. The effects of butyrate, SAHA and valproic acid on protein levels of c-Myc, Drosha and p57 were measured in HCT-116 cells using immunoblotting. Regulation of C13*orf*25 promoter activity by butyrate was analyzed by luciferase reporter assay using modified pGL3 constructs containing a wild-type or mutated c-Myc binding site. Expression of c-Myc was modulated using siRNA or adenovirus vectors. p57 mRNA and protein were measured before and after transfection with miR-92a-mimic molecules. Following butyrate treatment and miR-92a-mimic transfection, apoptosis was analyzed by TUNEL staining and caspase-3 immunoblotting.

**Results:**

Microarray, confirmed by qPCR, revealed a seven-fold increase in miR-92a levels in sporadic human colon cancer tissue compared to adjacent normal colon. Treating human colon cancer cells with butyrate reduced the levels of pri-miR17-92a, precursor and mature miR-92a, as well as c-Myc. SAHA and valproic acid had similar effects. Mutation of the c-Myc binding site diminished butyrate’s inhibitory effects on C13*orf*25 promoter activity. Silencing c-Myc expression reduced miR-92a levels. c-Myc over-expression neutralized butyrate-induced attenuation of pri-miR17-92a. Exogenous miR-92a inhibited butyrate-induced p57 expression and reversed the beneficial actions of butyrate on colon cancer cell proliferation and apoptosis.

**Conclusions:**

Our findings identify a novel cellular mechanism whereby butyrate inhibits miR-92a transcription by reducing c-Myc, thus augmenting p57 levels. These actions diminish colon cancer cell proliferation and stimulate apoptosis. This newly described regulation of oncogenic miRNA biogenesis expands our understanding of colon cancer cell biology and identifies novel therapeutic targets.

## Background

Disruption of the unique balance between dietary residue, intestinal flora and the host colonic mucosa that maintains intestinal homeostasis may trigger or promote diseases of the colon, including neoplasia. Short chain fatty acids (SCFAs) derived from dietary fiber by microbial anaerobic fermentation play an important role in this diet-microbiome-host interaction. Following the intake of dietary fiber, the major SCFAs, butyrate, proprionate, and acetate are produced and detected in both colonic luminal fluid and feces [1]. Epidemiological studies consistently identify a low-fiber diet, which reduces the bioavailability of butyrate, as a risk factor for colon cancer [2, 3]. Recent clinical studies also revealed that compared to controls, individuals with advanced colon cancer have diminished butyrate-producing bacteria and lower levels of SCFA [4, 5]. These findings suggest that compromised production of butyrate in the colon as a consequence of either low dietary fiber or diminished butyrate-producing bacteria promotes neoplasia.

Butyrate provides an important energy source for normal colon epithelial cells and promotes their proliferation [6]. By contrast, butyrate induces colon cancer cell apoptosis and differentiation, and inhibits proliferation [7]. It is likely that elucidating the mechanisms underlying these divergent actions of butyrate will identify candidate therapeutic targets. Proposed mechanisms include butyrate-induced transcriptional regulation as a histone deacetylase (HDAC) inhibitor, and inhibition of histone phosphorylation and DNA methylation, actions that suppress the expression of several oncogenes [8–10]. For example, by virtue of actions that accelerate mRNA degradation and inhibit transcript splicing, butyrate reduces expression of c-Myc, a key proto-oncogene [11–13]; butyrate treatment of human colon cancer cell lines reduces c-Myc mRNA and protein levels [14].

Two of the authors (SH, EBC) previously reported a novel mechanism involving modulation of cancer-associated miRNA profiles that mediates butyrate’s anti-cancer effects [15]. Among the miRNAs whose expression was suppressed by butyrate, members of the miR-106b family, including miR-17, miR-20a/b, miR-93 and miR-106a/b, regulate p21 translation and cancer cell proliferation [15, 16]. Colon cancer miRNA microarray data indicated that butyrate amplifies expression of other miRNA families and clusters including the oncogenic miR-17-92a cluster, also known as oncomiR-1 and C13*orf*25 [17, 18]. In addition to the aforementioned miR-17 and miR-20a, the miR-17-92a cluster encodes miR-18a, miR-19a/b and miR-92a. Targets of these miRNAs play critical roles in regulating pivotal cell processes including the cell cycle, proliferation, and apoptosis [19–23].

Here, we hypothesized that in colon cancer microbe-derived butyrate suppresses oncogenic miR-92a via regulation of c-Myc. Using both microarray and qPCR, we detected increased miR-92a levels in sporadic human colon cancer. Butyrate treatment of both HCT116 and HT29 human colon cancer cells reduced the levels of primary miR-17-92a (pri-miR-17-92a), precursor, and mature miR-92a; these effects were shared by other HDAC inhibitors (suberoylanilide hydroxamic acid (SAHA) and valproic acid). Butyrate treatment also decreased the levels of other miR-17-92a cluster members, including miR-17, miR-18a, miR-19a/b and miR-20a. Butyrate reduced c-Myc expression and c-Myc-induced miR-17-92a promoter activity. Silencing c-Myc protein expression reduced levels of miR-92a. In contrast, overexpressing c-Myc neutralized butyrate’s inhibitory effect on pri-miR-17-92a. Adding exogenous miR-92a reversed butyrate-induced p57 expression, growth inhibition, and apoptosis. Collectively, these findings uncovered a novel mechanism whereby interplay between butyrate and c-Myc regulates oncogenic miRNA biogenesis and promotes colon cancer cell apoptosis.

## Results

### Butyrate treatment diminishes miR-92a over-expression in human colon cancer cells

**Fig. 1:**
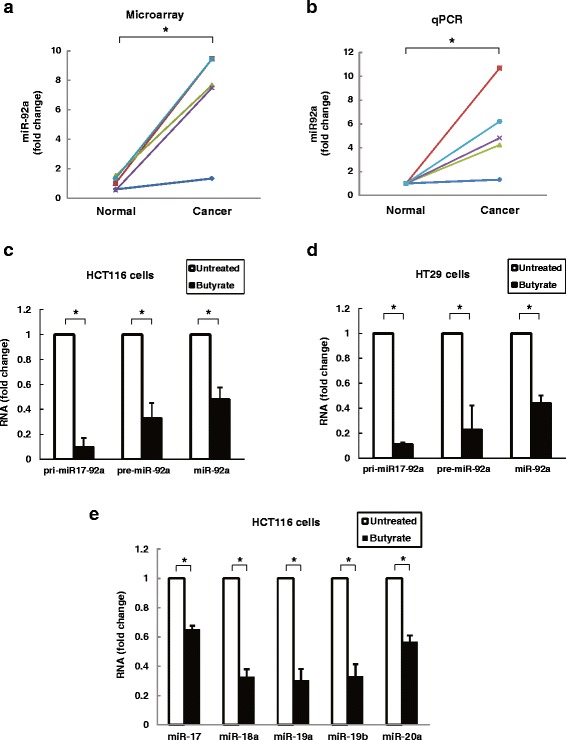
miR-92a is over-expressed in human colon cancer and its expression in human colon cancer cells is inhibited by butyrate treatment. **a** The abundance of miR-92a was measured in miRNA microarrays using RNA extracted from sporadic human colon cancer and adjacent normal tissue from the same five people. Data are shown as fold-change of sample to control reference pool signals. For purposes of direct comparison, samples from the same individual were labeled using the same color. *P < 0.05, *n* = 5. **b** Changes in miR-92a expression were confirmed by qPCR using the same tissue samples used for microarray. *P < 0.05, *n* = 5. Human colon cancer cells were treated with 2 mM butyrate for 24 h before extracting RNA. Untreated cells were used as control. The abundance of primary (pri-miR-17-92a), precursor (pre-miR-92a), and mature miR-92a was measured using qPCR in **c**) HCT116 and **d**) HT29. Pri-miR-106a-92a was not detected in either cell line. **e** The abundance of miR-17, miR-18a, miR-19a, miR-19b and miR-20a was measured in HCT116 cells. Bars represent means ± SEM. *P < 0.05, *n* = 4

The miR-17-92a cluster is over-expressed in a variety of cancers [17–19, 24, 25]. Previously, we showed that expression of several members of the miR-17-92a cluster is greatly augmented in human sporadic colon cancer [15]. As a first step to understanding how expression of this cluster is regulated, we used miRNA microarrays to analyze miR-92a expression in colon cancer compared to adjacent normal colon tissue from the same individual. As shown in Fig. 1a, in each of five paired samples we observed increased miR-92a expression in cancer compared to normal colon; mean miR-92a expression was seven-fold greater in the cancer samples. Using the same tissue samples, we confirmed these findings with qPCR (Fig. 1b).

Prior studies revealed that treating colon cancer cells with physiological dose of butyrate decreases expression of several miRNA clusters [15, 26]. We analyzed the effects of treating HCT116 and HT29 human colon cancer cells with the same dose (2 mM) of butyrate on miR-92a expression using qPCR to measure the abundance of primary (pri-miR-17-92a and pri-miR-106a-92a), precursor (pre-miR-92a), and mature miR-92a. Consistent with previous reports [21, 27], pri-miR106a-92a was not detected in human colon cancer cells. As shown in Fig. 1c, 24-h treatment of HCT116 cells with 2 mM butyrate down-regulated the levels of all pri-miR-17-92a, pre-miR-92a, and mature miR-92a. In response to butyrate treatment, we observed a 10-fold decrease in the initial miRNA transcript, pri-miR17-92a, and the levels of pre-miR-92a and mature miR-92a were decreased by 67 % and 52 %, respectively (Fig. 1c). Inhibition of pri-miR-17-92a, pre-miR-92a and miR-92a by butyrate was confirmed in a second human colon cancer cell line, HT29 (Fig. 1d). All members of the miR-17-92a cluster derived from pri-miR-17-92a were also measured in HCT-116 cells after butyrate treatment. As shown in Fig. 1e, consistent with previous reports [15, 26], miR-17, miR-18a, miR-19a/b and miR-20a, were decreased by 40 to 70 % after butyrate treatment. These findings suggested to us that the anti-neoplastic actions of butyrate might be mediated in part by the ability of butyrate to suppress oncogenic miRNA expression, particularly the miR-17-92a cluster.

### Effects of butyrate treatment on miR-92a and c-Myc expression

**Fig. 2:**
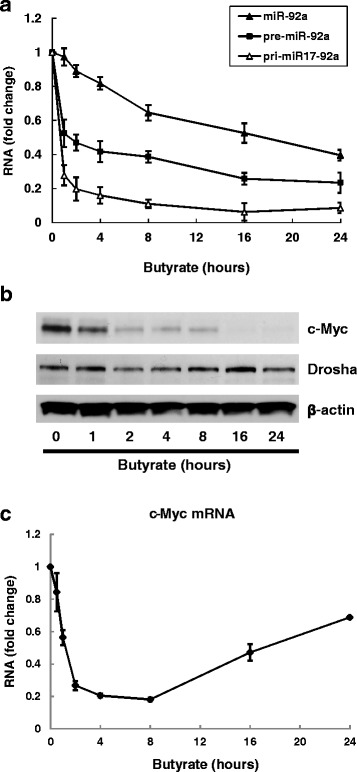
Time course of changes in expression of miR-92a, c-Myc, Drosha and p57 following butyrate treatment. HCT116 human colon cancer cells were treated with 2 mM butyrate for up to 24 h. Cells were harvested for analysis at 1, 2, 4, 8, 16, and 24 h after treatment. **a** The abundance of pri-miR-17-92a, pre-miR-92a, and mature miR-92a was measured using qPCR. Bars represent means ± SEM. *n* = 3. **b** Protein levels of c-Myc, Drosha and β-actin were analyzed by immunoblotting. The image shown is representative of three individual experiments. **c** The abundance of c-Myc mRNA was measured using qPCR. Bars represent means ± SEM. *n* = 3

To reveal the mechanism whereby butyrate regulates miR-92a expression, we examined the effects of butyrate treatment over 24 h in HCT116 cells. Pri-miR-17-92a, pre-miR-92a, and mature miR-92a were measured by qPCR 1, 2, 4, 8, 16, and 24 h after butyrate treatment (Fig. 2a). With butyrate treatment, mature miR-92a decreased gradually whereas pre-miR-92a and pri-miR-17-92a showed much steeper initial declines; pri-miR-17-92a levels in particular dropped sharply within the first 2 h after butyrate treatment.

In HCT116 cells using the same treatment conditions we measured protein and mRNA levels of c-Myc, a major transcription factor which up-regulates miR-17-92a cluster expression [18, 19, 23, 28]. As shown in Fig. 2b, substantial c-Myc protein was detected in untreated HCT116 cells but levels declined progressively over the course of 24 h of butyrate treatment; c-Myc expression declined ~70 % compared to basal values within 2 h of starting butyrate treatment. Neither Drosha, the key enzyme processing pri-miRNAs to pre-miRNAs, nor β-actin, a control housekeeping protein, were altered by butyrate treatment. Comparison of the time-courses for butyrate-induced attenuation of pri-miR-17-92a (Fig. 2a) and c-Myc (Fig. 2b) expression suggested to us that the actions of butyrate on miRNA transcription were most likely mediated by attenuation of c-Myc expression. c-Myc mRNA levels were rapidly reduced within the first 2 h of butyrate treatment, which correlated with change in c-Myc protein expression during the same time (Fig. 2c). Interestingly, after 8 h of butyrate treatment, we detected a gradual increase in c-Myc mRNA back to over 60 % of the basal level (Fig. 2c).

### Butyrate treatment inhibits C13*orf*25 promoter activity

**Fig. 3:**
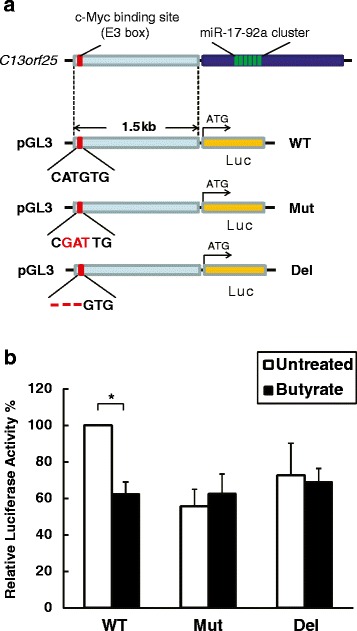
Requirement of the c-Myc binding site for the butyrate’s inhibitory effects on C13*orf*25 promoter activity. **a** Schematic of pGL3 luciferase reporter constructs containing the 1.5-kb promoter region upstream to the miR-17-92a cluster gene in C13*orf*25, which includes a wildtype functional c-Myc binding site (CATGTG, WT). The pGL3 vectors containing a mutated site (CGATTG, Mut) or a partially deleted site (− − − GTG, Del) were generated as controls. **b** Promoter activities of the three luciferase reporter constructs in response to butyrate treatment. Two hours after butyrate treatment (2 mM), HCT116 human colon cancer cells were transiently co-transfected with firefly luciferase pGL3 and pRL-TK Renilla luciferase vectors. Twelve h after transfection cells were harvested for measurement of luminescence. Cells without butyrate treatment (Untreated) were analyzed as control. Relative luciferase activity was normalized to the wild-type construct (Untreated). Bars represent means ± SEM. *P < 0.05, *n* = 4

A highly functional and conserved c-Myc binding site (E3 box, −CATGTG-) is located in the intronic C13*orf*25 promoter 1.5 kb upstream of the miR-17-92a coding sequence [19, 29]. We used a luciferase reporter system to investigate the effect of butyrate treatment on c-Myc-associated transcriptional regulation of the miR-17-92a cluster [30]. HCT-116 cells were transiently transfected with modified pGL3 vectors containing the 1.5-kb promoter segment of C13*orf*25 with either the wildtype c-Myc binding site (WT), a mutated site (Mut) or a partially deleted site (Del, see schematic in Fig. 3a). Firefly luciferase expression was used to assess the activity of the transcriptional promoter. The pRL-TK vector expressing Renilla luciferase was co-transfected to control for transfection efficiency. The WT construct showed substantial transcriptional activity under basal conditions (Untreated), and butyrate treatment significantly decreased luciferase activity by 40 % (Fig. 3b). Mutation or partial deletion of the c-Myc binding site attenuated promoter activity under basal conditions. Moreover, the inhibitory effect of butyrate was not detected in cells transfected with the mutated luciferase constructs (Fig. 3b). These reporter assay results suggested that the specific c-Myc binding site was required to mediate the inhibitory effect of butyrate on C13*orf*25 promoter activity.

### Silencing and over-expressing c-Myc alters pri-miR-17-92a expression

**Fig. 4:**
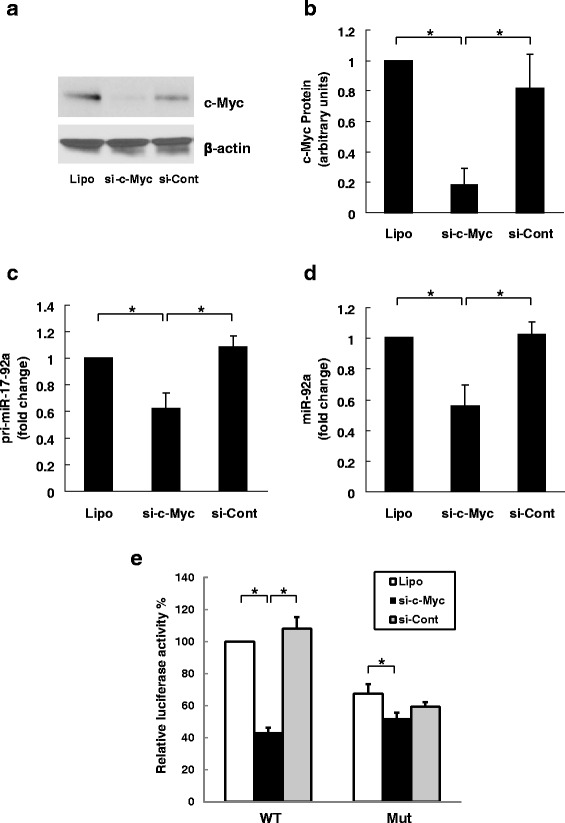
Effect of silencing c-Myc expression on miR-92a in HCT116 cells. HCT116 human colon cancer cells were transfected with predesigned MISSION siRNAs, an endoribonuclease-derived pool comprising a heterogeneous mixture of siRNAs targeting c-Myc mRNA (si-cMyc), or control siRNA (si-Cont) using Lipofectamine 2000 for 24 h before cell harvest. Cells treated with Lipofectamine 2000 alone (Lipo) served as control. **a** Protein levels of c-Myc and β-actin were analyzed by immunoblotting. The image shown is representative of four individual experiments. **b** Relative c-Myc protein levels were measured by densitometry. In HCT116 cells with control and reduced levels of c-Myc expression, qPCR was used to measure the abundance of **c** pri-miR-17-92a and **d** miR-92a. Bars represent means ± SEM. *P < 0.05, *n* = 4. **e** Eight hours after si-RNA transfection, HCT-116 cells were transiently co-transfected with pGL3 luciferase vectors containing wild-type (WT) or mutated (Mut) promoter region of miR-17-92a cluster as described in Fig. 3a and pRL-TK Renilla luciferase vectors. Twenty four hours after luciferase vector transfection, cells were harvested for measurement of luminescence. Relative luciferase activity was normalized to the wild-type control (Lipo). Bars represent means ± SEM. *P < 0.05, *n* = 4

To analyze further the regulatory effects of c-Myc and butyrate on pri-miR-17-92a transcription, we altered c-Myc protein levels in HCT116 cells using siRNA knockdown and adenovirus infection. To knockdown c-Myc, we transfected HCT116 cells with predesigned MISSION siRNAs, an endoribonuclease-derived pool comprising a heterogeneous mixture of siRNAs targeting c-Myc mRNA. As shown in Fig. 4a and b, c-Myc protein levels were substantially diminished after siRNA (si-cMyc) transfection whereas expression of the housekeeping protein β-actin was unchanged. c-Myc knockdown significantly reduced expression of pri-miR-17-92a (Fig. 4c) and mature miR-92 (Fig. 4d). Control siRNA (si-Cont) had no effect. As shown in Fig. 4e, using reporter assay with modified pGL3 constructs as described in Fig. 3a, we assessed the effects of silencing c-Myc in HCT-116 cells on pri-miR-17-92a promoter activity. Compared to basal (lipofectamine treatment only; Lipo), c-Myc knockdown reduced luciferase activity of the wild-type (WT) promoter by more than 55 %. In contrast, in cells expressing the mutated promoter (Mut) that had attenuated transcriptional activity, silencing c-Myc caused a much smaller change. Transfection with control siRNA (si-Cont) did not alter luciferase activity. These results show that maintaining high level c-Myc expression is essential for miR-17-92a transcription and that silencing c-Myc expression mimics the effects of butyrate treatment on miR-92a transcription.

**Fig. 5:**
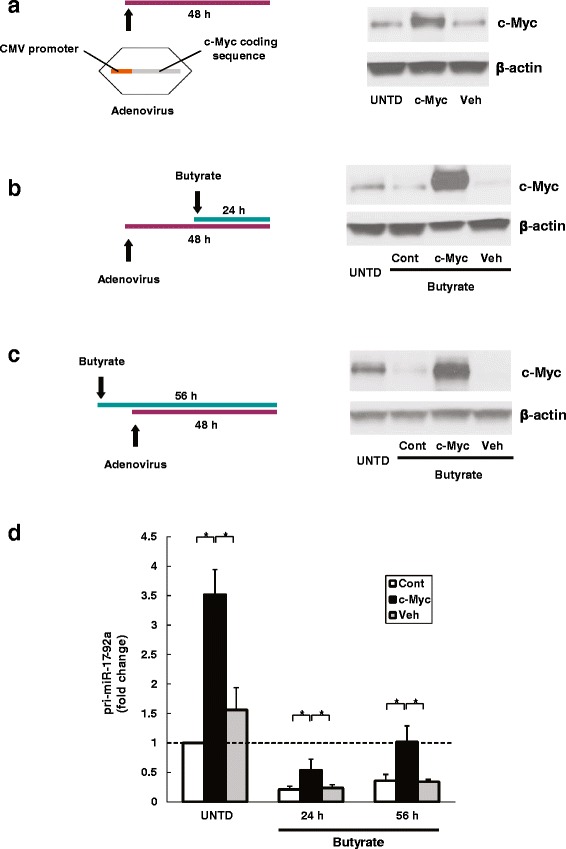
c-Myc-induced over-expression of pri-miR-17-92a was attenuated by butyrate treatment. HCT116 human colon cancer cells were infected with adenovirus carrying the c-Myc coding sequence driven by a CMV promoter to overexpress c-Myc (cMyc) or a null virus vehicle (Veh) for 48 h before analysis. Cells without virus infection were analyzed as control (Cont). The effects of treatment with 2 mM butyrate was assessed using three experimental designs: **a** no butyrate treatment, **b** 24-h treatment prior to cell harvest, and **c**) 56-h treatment starting 8 h prior to virus infection. Untreated cells (UNTD) were assessed as control. Protein levels of c-Myc and β-actin were analyzed by immunoblotting. Images shown are representative of three individual experiments. **d** The abundance of pri-miR-17-92a was measured using qPCR. Bars represent means ± SEM. *P < 0.05, *n* = 3

Next, we over-expressed c-Myc protein by infecting HCT116 cells with adenovirus carrying the c-Myc coding sequence driven by a CMV promoter (Fig. 5a schematic). After c-Myc-adenovirus infection, we confirmed increased c-Myc protein levels by immunoblotting cell extracts at 48 h, the optimal duration of treatment (Fig. 5a, right). c-Myc-adenovirus-infected cells had substantially higher pri-miR-17-92a levels compared to untreated cells (UNTD) and to control cells infected with null virus vehicles (Veh) (triad of bars on the left in Fig. 5d).

c-Myc-adenovirus-infected cells were incubated with 2 mM butyrate for 24 h before measuring changes in pri-miR-17-92a levels (Fig. 5b schematic). The immunoblot in the right panel of Fig. 5b shows that butyrate treatment diminished c-Myc protein levels in control cells (Cont and Veh) but due to its enhancement of CMV promoter activity butyrate treatment amplified c-Myc levels in c-Myc-adenovirus-infected cells. As shown by the middle triad of bars in Fig. 5d, pri-miR-17-92a levels were decreased in all butyrate-treated cells. Nonetheless, in c-Myc-adenovirus-infected cells reduction of pri-miR-17-92a levels was significantly attenuated compared to control cells (Cont and Veh); this finding suggested that c-Myc over-expression rescued cells from the butyrate-induced decrease in pri-miR-17-92a expression.

Parallel experiments were performed to analyze the effects of over-expressing c-Myc after 8 h of butyrate treatment (Fig. 5c schematic), a time point at which endogenous c-Myc protein and pri-miR-17-92a were depleted by butyrate treatment (Fig. 2). c-Myc-adenovirus infection robustly increased c-Myc expression (immunoblot in Fig. 5c), an effect likely due to prolonged (56 h) treatment with butyrate that enhanced CMV promoter activity. Also, pri-miR-17-92a levels were significantly greater in c-Myc-virus-infected compared to control cells (Cont and Veh; right triad of bars in Fig. 5d) but notably pri-miR-17-92a levels had recovered to nearly the same level as in untreated cells (UNTD in left triad of bars in Fig. 5d). From these experiments, we concluded that restoration of c-Myc expression in butyrate-treated cells rescues pri-miR-17-92a from inhibition.

### Effects of other HDAC inhibitors, SAHA and valproate, on miR-92a and c-Myc expression

**Fig. 6:**
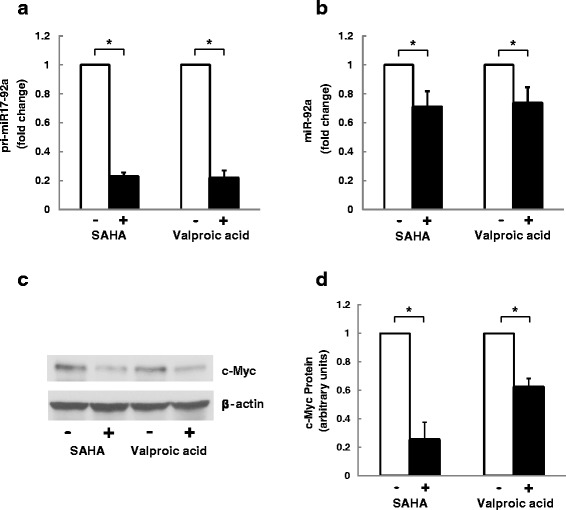
HDAC inhibitors, SAHA and valproic acid, suppressed expression of pri-miR-17-92a, miR-92a and c-Myc in colon cancer cells. HCT116 human colon cancer cells were treated with 0.5 uM SAHA or 1 mM valproic acid for 24 h before harvesting. The abundance of **a**) pri-miR-17-92a and **b**) miR-92a was measured using qPCR. Bars represent means ± SEM. *n* = 4. **c** Protein levels of c-Myc and β-actin were analyzed by immunoblotting. The image shown is representative of three individual experiments. **d** Relative c-Myc protein levels were measured by densitometry. Bars represent means ± SEM. *n* = 3

As a potent HDAC inhibitor, butyrate affects a broad range of gene expression by hyperacetylation of histones [9, 31]. In HCT-116 cells, we tested the effects of two other HDAC inhibitors, SAHA and valproic acid [32–34], on miR-92a and c-Myc expression in HCT-116 cells. As shown in Fig. 6a, we observed a 5-fold decrease in pri-miR-17-92a expression following treatment with 5 μM SAHA or 1 mM valproic acid for 24 h. SAHA or valproic acid treatment also down-regulated the level of mature miR-92a level of by ~30 % (Fig. 6b). Compared to basal values, c-Myc protein expression declined 75 % and 35 % after SAHA and valproic acid treatment, respectively (Fig. 6c and d). The control housekeeping protein, β-actin was not altered by these treatment.

### Transfection with exogenous miR-92a attenuates butyrate-stimulated p57 protein induction

**Fig. 7:**
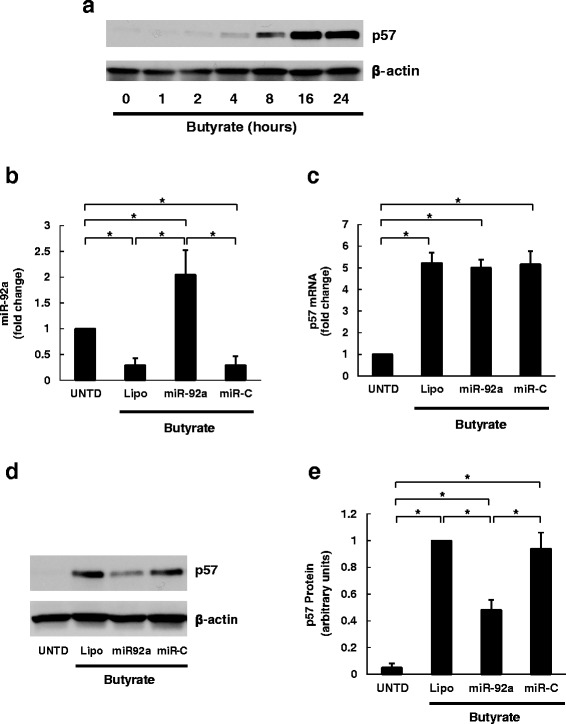
Over-expression of miR-92a attenuates butyrate-induced p57 expression by blocking p57 translation. **a** HCT116 human colon cancer cells were treated with 2 mM butyrate for up to 24 h. Cells were harvested for analysis at 1, 2, 4, 8, 16, and 24 h after treatment. Protein levels of p57and β-actin were analyzed by immunoblotting. The image shown is representative of three individual experiments. **b** HCT116 cells were transfected with miR-92a mimetics or control miRNA (miR-C) using Lipofectamine 2000 then treated with 2 mM butyrate for 24 h prior to harvest. Cells without butyrate treatment (UNTD) or treated with Lipofectamine 2000 (Lipo) were analyzed as controls. The abundance of miR-92a was measured using qPCR. **c** p57 mRNA levels were measured using qPCR. **d** Protein levels of p57 and β-actin were analyzed by immunoblotting. The image shown is representative of four individual experiments. **e** Normalized densitometric values of p57 protein levels. Bars represent means ± SEM. *P < 0.05, *n* = 4

Previous studies revealed a miR-92 target sequence in the 3′ UTR of p57, a tumor suppressor; miR-92 binding to this site inhibits p57 expression in a variety of cancers [16, 35, 36]. In Fig. 7a, we show that butyrate treatment induced progressive and robust p57 expression. To elucidate the mechanism underlying miR-92a effects on p57 expression, we transfected miR-92a-mimic molecules into HCT116 cells. As shown in Fig. 7b, transfection with miR-92a mimetics rescued cells from the effects of butyrate treatment, maintaining high levels of miR-92a. Butyrate induced a seven-fold increase in p57 mRNA levels, an effect unaltered by miR-92a mimetics (Fig. 7c). In contrast, butyrate induction of p57 protein levels was significantly attenuated by transfection with miR-92a mimetics (Figs. 7d and e). These results suggest that miR-92a inhibits p57 translation, not transcription.

### Anti-proliferative actions of butyrate are attenuated by over-expressing miR-92a

**Fig. 8:**
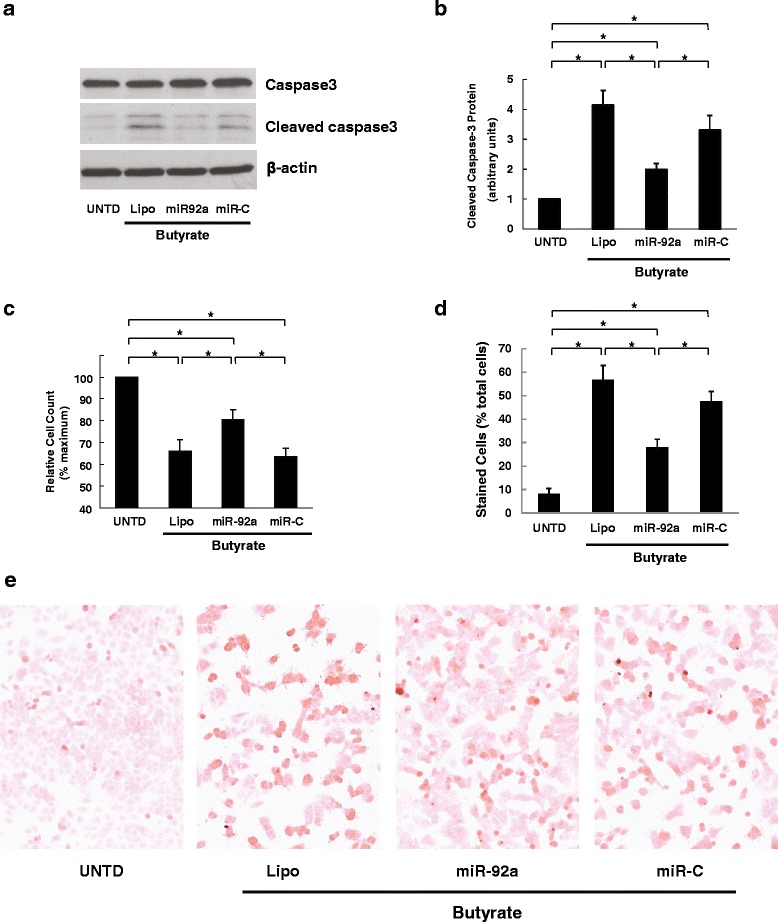
Over-expression of miR-92a reverses butyrate-induced effects on colon cancer cell proliferation and apoptosis. HCT116 human colon cancer cells were transfected with miR-92a mimetics or control miRNA (miR-C) using Lipofectamine 2000 and then treated with 2 mM butyrate for 24 h prior to harvest. Cells without butyrate treatment (UNTD) or treated with Lipofectamine 2000 (Lipo) were analyzed as controls. **a** Protein levels of total caspase-3, cleaved caspase-3 and β-actin were analyzed by immunoblotting. The image shown is representative of three individual experiments. **b** Normalized densitometric values of cleaved caspase-3 protein levels. **c** Relative cell count after miR-92a transfection and butyrate treatment. Cells were trypsinized and counted using a hemocytometer. Values shown are relative to the UNTD sample. **d** Percentage of positive cells in TUNEL staining. **e** The images of TUNEL staining shown are representative of three individual experiments. Positive cells stain brown. Results shown are means ± SEM. *P < 0.05, *n* = 3

p57 plays a critical role in various cancer cell functions, including proliferation and apoptosis [37]. Thus, miR-92a-dependent p57 induction may contribute to butyrate’s anti-cancer effects. To examine the role of miR-92a in mediating butyrate’s anti-proliferative and pro-apoptotic actions, prior to butyrate exposure HCT116 cells were transfected with exogenous miR-92a or control miRNA (miR-C), the same treatment as Fig. 7b-e. Butyrate-induced cleavage of caspase-3 was attenuated by exogenous miR-92a but not control miRNA (Fig. 8a and b). As shown in Fig. 8c, butyrate-induced reduction of HCT116 cell proliferation was significantly reversed by over-expressing miR-92a. Using a second apoptosis assay, TUNEL staining (Fig. 8d and e), we confirmed that transfection with miR-92a reduced the proportion of butyrate-induced, TUNEL-positive apoptotic cells. Again, butyrate-induced inhibition of colon cancer cell proliferation was attenuated by adding miR-92a but not control miRNA (Fig. 8e). Collectively, these findings support a key role for miR-92a in mediating the functional effects of butyrate treatment on colon cancer cells.

## Discussion

The present study identifies a novel regulatory mechanism whereby butyrate inhibits miR-92a biogenesis. As a microbe-derived by-product of fiber fermentation, butyrate plays a critical role in modulating host gene expression in the colon. Previously, we showed that multiple members of the miR-17-92a cluster were greatly suppressed by treating human colon cancer cells with butyrate [15]. In this study, we focused on elucidating the mechanism whereby butyrate regulates biogenesis of the oncogenic miR-17-92a cluster. In HCT116 and HT29 human colon cancer cells, butyrate treatment reduced miR92a levels at all processing stages, amongst which the initial pri-miR-17-92a transcripts showed the most rapid and largest declines after butyrate treatment. All members of the miR-17-92a cluster derived from pri-miR-17-92a were decreased by butyrate in HCT 116 cells. These results suggested that butyrate’s effects on miR92a inhibition were likely mediated by transcriptional regulation of pri-miR-17-92a and less likely a consequence of altered miRNA processing.

The transcriptional activity of the miR-17-92 cluster originates from the core promoter region directly upstream of the miRNA coding sequences. This site includes multiple transcription factor binding sites [19, 20, 29, 38]. Transcription of the miR-17-92 cluster is strongly dependent on c-Myc binding to a conserved E-box element (E3) in the core promoter region [18, 23]. In K562 myelogenous leukemia and HeLa cervical cancer cells, silencing c-Myc protein expression or deleting the E3 element significantly reduced transcriptional activity [30].

We found that butyrate treatment greatly diminished c-Myc protein levels in colon cancer cells, an action that resulted in suppression of C12*orf*25 promoter activity and miRNA transcription. Silencing c-Myc in HCT116 cells decreased C12*orf*25 promoter activity and levels of both pri-miR-17-92a and mature miR92a, thus mimicking the actions of butyrate. Over-expressing c-Myc resulted in up-regulated levels of pri-miR-17-92a. Butyrate enhances CMV promoter activity [39], therefore exogenous c-Myc gene maintained robust expression even after butyrate treatment. Pre-existing high levels of c-Myc protein partially blocked the suppression of pri-miR-17-92a by butyrate treatment. In rescue experiments, restoring butyrate-suppressed c-Myc protein levels fully restored pri-miR-17-92a to baseline levels. In concert, these findings are compatible with a mechanism whereby butyrate-induced attenuation of c-Myc expression regulates miRNA levels.

Humphrey et al. confirmed butyrate’s actions in reducing levels of members of the miR-17-92a cluster in colon cancer cells [26]. In the same study, although levels of c-Myc mRNA were paradoxically increased after butyrate treatment, c-Myc protein levels were not measured; the c-Myc-mediated regulatory mechanism was not pursued. Reduced c-Myc expression after butyrate treatment is reported in several colon cancer cell lines [11, 14]. Various causative mechanisms were implicated, including suppression of mRNA transcription, accelerated degradation of c-Myc mRNA, and inhibited splicing of c-Myc transcripts [12]. In the present study, a rapid decrease was noted in c-Myc protein and mRNA levels within the first 2 h of butyrate treatment. However, c-Myc mRNA re-accumulated after 8 h of butyrate treatment without restoring the level of c-Myc protein, this likely represents negative transcriptional feedback in response to diminished c-Myc protein. A mechanism of translational inhibition may underlie this mismatch of c-Myc mRNA and protein, resulting in paradoxically elevated c-Myc mRNA levels after butyrate treatment as observed by Humphrey et al. [26].

As a consequence of the network of post-transcriptional processing, including nuclear processing by Drosha/DGCR8, nuclear export, cytoplasmic processing by dicer, and RNA degradation of primary, precursor and mature miRNAs [40–43], the complexity of miRNA biogenesis surpasses that for classical mRNA. Thus, although our findings implicate c-Myc in mediating the effects of butyrate on pri-miR-17-92a transcription, we cannot exclude a role for other regulatory mechanisms, including c-Myc-independent inhibition of pri-miRNA transcription or pri-miRNA degradation by butyrate. However, because all primary, precursor, and mature miRNAs decreased in a gradient fashion with the largest changes seen on pri-miR-17-92a, mechanisms regulating miRNA processing in the nucleus and cytosol are less likely to be affected by butyrate. Moreover, expression of Drosha, a major player in processing primary to precursor miRNAs, was unchanged by butyrate treatment (Fig. 2b).

Altered miRNA expression can influence cancer development when miRNA targets are tumor suppressors or oncogenes. In the colon, up-regulated miR-17-92a promotes neoplasia through various pathways, e.g. miR-18a and miR-19 directly repress TSP-1 and CTGF, respectively, to promote angiogenesis [24] and miR-92a down-regulates BCL2L11 expression thereby reducing apoptosis [44]. Previously, we reported that butyrate decreased miR-17 and miR20a levels in HCT116 cells, thereby allowing p21 expression to down-regulate cell proliferation [15].

**Fig. 9:**
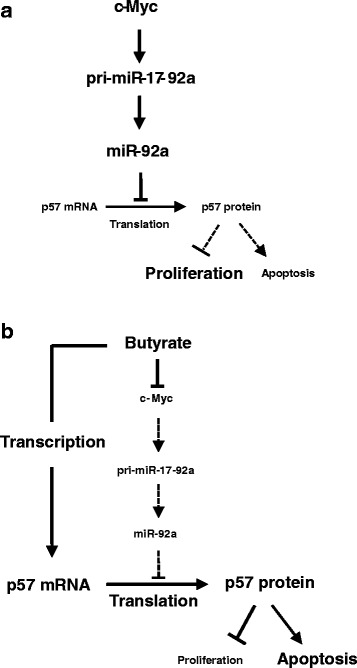
Butyrate decreases c-Myc and miR-92a levels and increases p57 expression in colon cancer cells. **a** In colon cancer cells, high levels of c-Myc up-regulate miR-17-92a *transcription*. Over-expressed miR-92a inhibits p57 mRNA translation, thus attenuating the cellular actions of p57 protein. **b** Treatment with butyrate up-regulates both p57 *transcription* and p57 mRNA *translation*. The effect of butyrate on p57 mRNA translation is mediated via reduced c-Myc protein expression, which decreases miR-17-92a cluster transcription and miR-92a levels. Collectively, the interactions between butyrate, c-Myc, miR-92a and p57 mediate butyrate’s anti-proliferative and pro-apoptotic effects, both relevant to colon neoplasia

In the present study, we demonstrate a parallel pathway involving miR-92a and p57 that contributes to butyrate’s anti-cancer effects (Fig. 9). p57 modulates many cellular processes that are dysregulated in cancer, including cell cycle control, differentiation, apoptosis, and development [37]. Often, p57 is epigenetically silenced in cancer [45, 46], partially due to transcriptional regulation by histone deaceylation/methylation and translational regulation by miRNAs such as miR-221/222, miR-25, miR-92b and miR-92a (Fig. 9a) [16, 35, 47–49]. In human colon cancer cells, butyrate and other HDAC inhibitors up-regulate p57 levels by enhancing mRNA *transcription* (Fig. 9b) [50, 51]. Simultaneously, butyrate decreases miR92a levels, thereby permitting p57 mRNA *translation* (Fig. 9b). Based on microarray result from a previous study [15], butyrate also down-regulates expression of miR-221/222 and miR-25. For example, miR-25 levels decreased ~30 % after butyrate treatment, confirmed by qPCR (data not shown). Decreased expression of miRNAs that share the same targeting sequence in the p57 3’UTR may synergistically regulate p57 expression.

## Conclusions

The actions of butyrate exemplify interactions between diet, bacteria, and host epithelial cells that are critical to maintaining gut homeostasis. Compromised butyrate production due to interruption of this balance has been implicated repeatedly in colon neoplasia. Here, we identified a novel mechanism whereby butyrate, a HDAC inhibitor, regulates oncogenic miRNA biogenesis via c-Myc to attenuate human colon cancer cell proliferation and promote apoptosis (Fig. 9). This mechanism whereby diet-microbial interaction regulates host gene expression expands our understanding of colon neoplasia. It is anticipated that further unraveling the mechanisms underlying butyrate’s beneficial effects will identify new therapeutic strategies to prevent and treat colon cancer.

## Methods

### Human tissues

Tissue samples were obtained from individuals with colon cancer at the University of Chicago Medical Center under a protocol approved by the Institutional Review Board. Prior to tissue collection, informed consent was obtained from each subject. All clinical investigations using human subjects were conducted according to the principles expressed in the Declaration of Helsinki. At surgery, tissue was obtained from colon cancer and adjacent normal-appearing mucosa (more than 5 cm from the tumor border). These tissue samples were immediately rinsed in ice-cold phosphate-buffered saline (PBS) before cell lysis for RNA extraction.

### MicroRNA microarray

Total RNA was extracted from human colon samples using the mirVana miRNA Isolation Kit (Ambion, Life Technologies) according to the manufacturer’s protocol. Human miRNAs were analyzed using mirVana miRNA Bioarrays v. 2 (Ambion), which utilized the miRBase sequence database (version 8.0). RNA samples were labeled with the mirVana miRNA labeling kit and hybridized to miRNA bioarrays per product instructions. The arrays were scanned using a GenePix4000B fluorescence scanner in the University of Chicago Functional Genomics Core Facility. Five pairs of colon tissue samples were analyzed.

### Cell culture

Human colon cancer cell lines, HCT116 (male, CCL-247) and HT29 (female, HTB-38), were authenticated and acquired from American Type Culture Collection (ATCC). Cells were grown at 37 °C in Modified McCoy’s 5a Medium (Life Technologies) containing 10 % fetal bovine serum, 50 U/ml penicillin and 50 mg/ml streptomycin. Cells were rinsed, scraped and pelleted in ice-cold PBS for protein and RNA extraction. Sodium butyrate (B5887, Sigma-Aldrich) was added to culture media at a final concentration of 2 mM. SAHA (SML0061, Sigma-Aldrich) dissolved in DMSO was added to the culture media at a final concentration of 5 μM. Cells treated with 0.1 % DMSO were analyzed as control. Sodium valproate (P4543, Sigma-Aldrich) was added to the culture media at a final concentration of 1 mM.

### Immunoblotting

Pelleted cells were homogenized in lysis buffer containing 20 mM Tris (pH 7.5), 100 mM NaCl, 5 mM MgCl_2_, 1 mM EDTA, 1 % Triton X-100, 1 mM sodium fluoride, 1 mM sodium vanadate, 1 mM phenylmethylsulfonyl fluoride, 1 μg/ml pepstatin, and 1 μg/ml leupeptin. Total protein was quantified using the BCA protein assay (Thermo Scientific). Protein samples (20 μg) solubilized in 2X Laemmli buffer were separated by SDS-PAGE and transferred to polyvinylidene difluoride (PVDF) membranes. Membranes were blocked with 5 % wt/vol non-fat dry milk in Tween-Tris buffered saline (TTBS). Primary antibodies, specific for c-Myc, Drosha, p57, caspase-3, cleaved caspase-3 and β-actin (Cell Signaling), were added and incubated overnight at 4 °C. Membranes were incubated with horseradish peroxidase-conjugated species-appropriate secondary antibodies (Cell Signaling) for 1 h at room temperature, and developed using a Western Lightning *Plus* ECL kit (Perkin Elmer). Image quantification was performed by scanning densitometry using NIH Image J 1.54 software.

### Quantitative real-time PCR (qPCR) for precursor and mature miRNAs

Total RNA was extracted from pelleted cells by Trizol (Life Technologies) according to the manufacturer’s instructions. Complementary DNA was synthesized from total RNA samples using the NCode Vilo miRNA cDNA Synthesis Kit (Life Technologies). Real-time PCR was performed with ABI StepOnePlus real-time PCR system (Applied Biosystems) using Veriquest Sybr Green qPCR Master (Affymetrix) with miRNA-specific primers and a universal qPCR primer according to the manufacturer’s protocol for the NCode VILO Kit. The two-step quantification cycling protocol (2 min at 50 °C, 10 min at 95 °C and then 40 cycles of 95 °C for 15 s and 60 °C for 60 s) was used. PCR specificity was confirmed by melting curve analysis. All miRNAs were normalized to a small nucleolar RNA, *RNU48* [52]. Primers used were *miR-92a 5’*- TATTGCACTTGTCCCGGCCTGT -3’; *pre-miR-92a* 5’- CTTTCTACACAGGTTGGGATCG -3’; and *RNU48* 5’- GATGACCCCAGGTAACTCTGAG -3’; *miR-17* 5’- CAAAGTGCTTACAGTGCAGGTAG -3’; *miR-18a* 5’- TAAGGTGCATCTAGTGCAGATAG -3’; *miR-19a* 5’- TGTGCAAATCTATGCAAAACTGA -3’; *miR-19b* 5’- TGTGCAAATCCATGCAAAACTGA -3’; *miR-20a* 5’- TAAAGTGCTTATAGTGCAGGTAG -3’. For quantification, the fold-change of miRNA in experimental relative to control samples was determined by the 2^-∆∆Ct^ method [53].

### Quantitative real-time PCR for pri-miRNAs and mRNAs

After total RNA extraction, complementary DNA was synthesized using SuperScript III (Life Technologies) and a random hexonucleotide primer. The sense and antisense PCR primers used for real-time PCR for primary miRNAs and mRNAs were *p57*: 5’- CCATCTAGCTTGCAGTCTCTTC -3’ and 5’- GACGGCTCAGGAACCATTT -3’; *GAPDH*: 5’- CTCCTCACAGTTGCCATGTA -3’ and 5’- GTTGAGCACAGGGTACTTTATTG -3’; *c-Myc*: 5’- CATACATCCTGTCCGTCCAAG -3’ and 5’- GAGTTCCGTAGCTGTTCAAGT -3’; *pri-miR-17-92a*: 5’- AGTGAAGGCACTTGTAGCATTA -3’ and 5’- GCACTAGATGCACCTTAGAACA -3’; *pri-miR-106a-92a*: 5’- GAGAGGGGGAGTCCAAAATC -3’ and 5’- TGGTTTCAACCAAATCCTGA -3’. All pri-miRNAs and mRNAs were normalized to GAPDH.

### Cell transfection

Lipofectamine 2000 (Life Technologies) was used to transfect luciferase plasmids, silencing RNA or miRNA molecules. Pre-designed MISSION siRNAs specific to human c-Myc (esiRNA1, Sigma-Aldrich) were used to knock down c-Myc expression. MISSION siRNAs are endoribonuclease-prepared siRNA pools comprised of a heterogeneous mixture of siRNAs that all target the same mRNA sequence. Cells were transfected with siRNAs for 48 h prior to harvest for protein or RNA extraction. To overexpress miR-92a, an engineered miR-92a mimetic molecule (Ambion’s Pre-mir MiRNA Precursor Molecules) was used to transfect HCT116 cells according to the manufacturer’s protocol. miR-C (Ambion) was used as a control.

### Luciferase reporter assay

Modified pGL3 constructs with C13*orf*25 promoter segments upstream of the firefly luciferase coding sequence (Fig. 3a) were a generous gift from Dr. Grünweller, Institute of Pharmaceutical Chemistry, Philipps University Marburg, Germany [30]. A point mutation and partial deletion of the c-Myc binding E3 element (Fig. 3a) were introduced to the luciferase constructs using QuikChange XL Site-Directed Mutagenesis Kit (#200516, Agilent Technologies) according to the manufacturer’s protocol. Two h after butyrate treatment, HCT-116 cells were transiently co-transfected with modified pGL3 constructs and pRL-TK plasmids (E2241, Promega) in a 10:1 ratio using Lipofectamine 2000. Twelve h after transfection, cells were harvested by shaking in lysis buffer (Promega). Firefly and Renilla luciferase activities in the lysate were determined with a Dual-Luciferase Reporter assay system (Promega) according to the manufacturer’s instructions. As a transfection efficiency control, firefly luciferase activity was normalized to Renilla luciferase activity.

### Recombinant viral infection

Replication-defective adenovirus was used to over-express c-Myc as described previously [54]. Recombinant adenoviral plasmids derived from the Adeno-X Expression System (Clontech) containing full length human c-Myc gene were transfected into HEK-293 cells for adenoviral particle packaging. pAdeno-X, the recombinant replication-incompetent adenovirus carrying no c-Myc cDNA insert, was grown and served as a control adenovirus (null virus vehicle). The potency of adenovirus was titrated as previously described . For infection, virus particles were added to culture media of HCT116 cells. c-Myc expression was assessed 48 h after infection.

### TUNEL staining

Terminal deoxynucleotidyl transferase dUTP nick end labeling (TUNEL) staining for apoptosis analysis was performed using the DeadEnd colorimetric TUNEL system (Promega) according to the manufacturer’s protocol. Briefly, after 24-h butyrate treatment, HCT116 cells on glass slides were fixed and permeabilized. After equilibration, cells were incubated in rTdT reaction mix for 60 min at 37 °C. Cells were blocked in 0.3 % hydrogen peroxide and then developed in DAB solution. All images were acquired using the Nikon Eclipse 80i microscope system and Image-Pro Plus 5.1 software with standard image processing. To calculate the percentage of stained cells, 200 cells were counted in five microscopic fields on every slide.

### Statistical analysis

Results are presented as means ± SEM for the indicated number of experiments. The results of multiple experiments were analyzed by Student’s *t*-test or ANOVA using the Bonferroni correction for multiple comparisons.
